# Cheap and Commonplace: Making the Case for BCG and γδ T Cells in COVID-19

**DOI:** 10.3389/fimmu.2021.743924

**Published:** 2021-09-08

**Authors:** Alexandra L. Morrison, Sally Sharpe, Andrew D. White, Mark Bodman-Smith

**Affiliations:** ^1^Public Health England, National Infection Service, Porton Down, United Kingdom; ^2^Infection and Immunity Research Institute, St George’s University of London, London, United Kingdom

**Keywords:** gamma delta T cell, Bacille Calmette-Guérin vaccine, trained immunity, non-specific immunity, COVID-19, innate immunity, vaccine, antiviral

## Abstract

Antigen-specific vaccines developed for the COVID-19 pandemic demonstrate a remarkable achievement and are currently being used in high income countries with much success. However, new SARS-CoV-2 variants are threatening this success *via* mutations that lessen the efficacy of antigen-specific antibodies. One simple approach to assisting with this issue is focusing on strategies that build on the non-specific protection afforded by the innate immune response. The BCG vaccine has been shown to provide broad protection beyond tuberculosis disease, including against respiratory viruses, and ongoing studies are investigating its efficacy as a tool against SARS-CoV-2. Gamma delta (γδ) T cells, particularly the Vδ2 subtype, undergo rapid expansion after BCG vaccination due to MHC-independent mechanisms. Consequently, γδ T cells can produce diverse defenses against virally infected cells, including direct cytotoxicity, death receptor ligands, and pro-inflammatory cytokines. They can also assist in stimulating the adaptive immune system. BCG is affordable, commonplace and non-specific, and therefore could be a useful tool to initiate innate protection against new SARS-CoV-2 variants. However, considerations must also be made to BCG vaccine supply and the prioritization of countries where it is most needed to combat tuberculosis first and foremost.

## Introduction

In January 2020 the WHO declared Coronavirus disease 19 (COVID-19) a Public Health Emergency of International Concern (PHEIC), and a pandemic in March 2020. As of July 2021 this virus is responsible for nearly four million deaths worldwide (1). COVID-19 represents a broad spectrum of clinical syndromes, from asymptomatic disease, mild flu-like symptoms, to severe pneumonia and acute respiratory distress syndrome (ARDS). Safe and effective vaccines have now been developed to combat COVID-19 spread. However, the highly specific nature of these vaccines leaves them susceptible to escape mutations. This, along with additional concerns around supply, especially in low- and middle-income countries (LMICs), justifies the search for common, affordable and non-specific strategies to be used in combination with specific vaccines or as an interim measure. Here we make the case for the Bacille Calmette-Guerin (BCG) vaccine and its role in stimulating gamma delta (γδ) T cells, particularly the Vδ2 subset.

The causative agent of COVID-19 is severe acute respiratory syndrome coronavirus 2 (SARS-CoV-2). SARS-CoV-2 is a positive sense single stranded RNA virus, able to spread between humans in close contact, *via* respiratory droplets produced from coughs and sneezes, and probable fomites. The virus is able to enter the respiratory epithelial cells of the oropharynx and upper airway *via* its spike glycoprotein, which targets the angiotensin converting enzyme 2 (ACE2) receptor. Binding causes conformational changes in the spike protein, mediating the fusion of the viral and cell membranes and the release of the viral nucleocapsid into the cell (2). Part of the reason SARS-CoV-2 is more transmissible than SARS-CoV is because of structural differences on its surface proteins that allow stronger binding to the ACE2 receptor (3, 4).

SARS-CoV-2 variants are now being identified that have a multitude of further mutations that allow even stronger binding of the ACE2 receptor, and therefore are spread even more easily. An example of this is the N501Y mutation, present in the Alpha variant, which alters an amino acid within the six key residues in the receptor biding domain of the spike glycoprotein, which has arisen independently in various locations including the UK, South Africa and Australia (5). It has been shown that additional mutations may result in lessened antibody effectiveness (6), and there is growing concern around variants rendering existing vaccines less efficacious. The current principal variant of concern in the UK, the Delta variant, contains mutations in the spike protein, including E484K and L452R, that, in addition to strengthening ACE2 receptor binding, can reduce the ability of vaccine stimulated antibodies to attach to the altered spike protein (7, 8). In light of these concerns, vaccine strategies that are able to offer a broader base of protection, and therefore are more resistant to mutations than single target strategies, could prove an important additional tool in our arsenal against SARS-CoV-2 variants.

Vaccines against SARS-CoV-2 including those manufactured by Pfizer, Moderna and AstraZeneca, are currently being used in wealthy nations with great success. However, with production limited and demand greatly exceeding supply, it may be some years before LMICs are able to complete their own nationwide COVID-19 vaccination programs. This vaccine inequality only enhances opportunities for additional mutations to arise that further reduce vaccine protection. Continued research into additional strategies that could be used in conjunction with SARS-CoV-2 antigen specific vaccines to combat COVID-19 is needed.

BCG is the most widely used vaccine in the world, and in recent years has been used most extensively in LMICs. When it was first introduced to Europe in the 1920s it was observed that vaccination provided non-specific, otherwise known as off-target, protection against a range of diseases, particularly respiratory infections (9). Since the SARS-CoV-2 pandemic there have been many observational studies reporting a level of protection in BCG vaccinated adults and children (10–12). An ecological study found both cases and deaths in countries with national BCG vaccination programs were significantly lower in March 2020 than in countries without (10). Escobar et al., found that with every 10% increase in BCG index (an estimation of vaccination coverage) there was a corresponding 10.4% decrease in COVID-19 deaths (11). Additionally, in Japan, prefectures with higher BCG vaccine coverage had fewer COVID-19 infections (12). However, another study in Sweden looked at people born just before or just after 1975, when universal BCG vaccination ceased, and did not find any statistically significant difference in COVID-19 cases and hospitalizations (13). Twelve randomized control trials (RCTs) studying BCG and COVID-19 are presently underway in various countries, although results from most of these studies are still many months away. However, the findings from one randomized trial are now available in preprint; the ACTIVATE-2 study, which revaccinated elderly Greek patients with BCG, found a reduction in COVID-19 clinical and microbiological diagnoses compared to the placebo group (14).

Recent articles have outlined how BCG is able to reprogram the innate system, resulting in an altered innate immune response to subsequent infections (15). This so-called ‘training’ of innate immune cells, which includes epigenetic, transcriptional, and functional reprogramming, is thought to be largely responsible for much of the off-target beneficial impact of BCG on non-tuberculosis diseases, including viral diseases. The pathways impacted by trained immunity include those that may be important for the control of COVID-19 disease, as reviewed by others (16–22).

Much is now known about BCG and its ‘training’ of innate cells, but less is known about the role of γδ T cells in this non-specific action. γδ T cells, of which Vδ1 and Vδ2 cells are the main subtypes in humans, are unconventional T cells that bridge the innate and adaptive immune system. They have been shown to be a significant component of the early innate immune response to many viral infections. Importantly, Vδ2 T cells proliferate rapidly after BCG stimulation, as well as being one of the main producers of IFN-γ in this vaccination response. Studies have also shown they demonstrate recall responses. These long lasting, memory-like responses, which include rapid production of proinflammatory cytokines and cytotoxic granules essential for viral clearance (23), indicate γδ T cells might be a key player in BCG non-specific protection to viruses, including SARS-CoV-2.

## The Heterogenous Effects of BCG Vaccination

BCG is an attenuated form of *Mycobacterium bovis* which has been used in humans as a tuberculosis (TB) vaccine since the 1920s. BCG remains to this day a critical component of the strategy to combat TB, with the focus on vaccinating infants shortly after birth in endemic areas. Although there is a high efficacy against childhood TB (24), protection wanes with age, and the efficacy of adult BCG vaccination varies widely in different studies from 0 – 80% (25). Revaccinating in adolescence has been proposed as one way to boost this protection, with Nemes et al. demonstrating revaccination reduced the rate of sustained QuantiFERON TB Gold InTube (QFT)-conversion, reflecting better bacterial control and clearance (26). The REVAX clinical trial is ongoing to assess whether revaccination of adolescence may be a useful tool for TB control.

After BCG was introduced in the 1920s, epidemiological studies reported that BCG vaccination greatly reduced infant mortality, beyond that which could be explained by a reduction in TB alone (9). These observations were confirmed by RCT studies, including one showing that giving BCG to low birth weight children could reduce mortality by 50% (27). The reduction in mortality was mostly from respiratory infections, which are for the most part viral, and sepsis. Another recent RCT study found BCG can protect the elderly against respiratory infections (14). Observational studies looking at BCG in humans have demonstrated protective roles for BCG in syncytial virus infection (28); respiratory tract infections and pneumonia in older individuals (29, 30); and yellow fever (31). This non-specific protective role in viral infections has also been demonstrated in vaccinated mice, where studies as early as the 1970s showed BCG vaccination reduced influenza virus titer (32), and provided a level of protection against herpes simplex virus (HSV) (33). Another study found that even the administration of just components of the mycobacterial cell wall was enough to provide some protection against vaccinia virus and herpes simplex virus 2 (HSV2) (34). Now studies are showing a similar effect with COVID-19, with one retrospective cohort study finding an association between BCG vaccine in the five years prior and a lower incidence of sickness and extreme fatigue during the COVID-19 pandemic (35). Where BCG can be used on its own to stimulate innate immunity, it has also successfully been used as an adjuvant in more specific vaccine strategies against SAS-CoV-2 infection (36).

The non-specific protection afforded by BCG is often referred to as ‘trained immunity’. Although much is still uncertain regarding how this protection comes about, it is now known to involve long-lasting changes in cells of the innate immune system, including monocytes, macrophages, dendritic cells (DCs), mucosal associated invariant T (MAIT) cells, natural killer (NK) cells and γδ T cells. Most innate cells were previously believed to be static and unchanged after encountering stimuli (37), and therefore investigations into trained immunity have resulted in a shift of central immune system dogma. The changes that result in the non-specific protection BCG provides against many viral infections are likely a combination of epigenetic, transcriptional, and functional reprogramming, as well as the induction of memory-like cells (15, 38).

Epigenetic changes after BCG include the upregulation of innate cell transcripts in the bone marrow of hematopoietic cells, as well as inducing greater DNA-accessibility around genes associated with inflammation in existing innate cells (39). Chemical modifications (methylation and acetylation of histones) allow for greater accessibility of chromatin, and easier transcription of genes (40). This results in the rapid and sustained upregulation of antimicrobial responses in innate cells upon subsequent infection, of which monocytes and NK cells are the most characterized. Kleinnijenhuis et al., demonstrated that macrophages isolated from BCG vaccinated healthy adults showed enhanced production of the pro-inflammatory cytokines IL-1β, TNF-α and IL-6 when stimulated *ex vivo* with unrelated bacterial and fungal antigens (41). Similar findings have been seen in against viruses, with BCG vaccination inducing greater protection against attenuated yellow fever virus vaccine strain, which correlated with an increase in the upregulation of IL-1β (31). A further RCT in Ugandan infants found that just delaying BCG vaccination from birth to six weeks old, significantly increased infectious disease incidence. They found the protection afforded by BCG was related to histone trimethylation at the promoter region of pro-inflammatory cytokines, including TNF and IL6, indicating immune cells were primed for pro-inflammatory responses (42). Specifically, monocytes show a particular increase in H3K4me3 histone modification, involved in transcriptional activation of TLR4, TNFα, and IL6 genes (43, 44)

These responses are also longer lasting than initially thought possible by innate cells. BCG trained monocytes were identified in the blood three months after vaccination, when their normal half-life in circulation may only be up to one day (39). Both NK and γδ T cells have been shown to exhibit memory-like properties after BCG vaccination, that are sustained for several months (38). The memory phenotype of γδ T cells induced in response to BCG was observed by Hoft et al., in 1998, after PBMCs from BCG vaccinated humans were cultured with mycobacterial antigens. Seven days later the cell type that had undergone the greatest expansion in comparison to cells from unvaccinated control cultures was the γδ T cell (45). Primate studies demonstrated the occurrence of a recall expansion by γδ T cells after *Mycobacterial tuberculosis* (*M. tb*) infection, and the kinetics of the recall expansion was dissimilar to the *M. tb* primary expansion (46). This recall expansion coincided with protective immunity. Recently the expansion of Vδ2 T cells after BCG was confirmed in humans *in vivo* as well as the production of IFN-γ by Vδ2 T cells after vaccination (47). Interestingly, other donor unrestricted T (DURT) cells, such as MAIT and NK cells were not altered after BCG vaccination or revaccination in humans in this study.

## Gamma Delta T Cells

γδ T cells are important players in the early immune response to infections or malignant transformation, as well as being involved in the adaptive response. γδ T cells are powerful effector cells, despite only representing 0.5-5% of circulating T cells in homeostatic conditions (48). Their numbers rapidly expand in the circulation in response to stimuli due to the non-MHC restricted recognition of unprocessed antigens. γδ T cells also represent a much higher proportion of immune cells at barrier surfaces such as mucosal and epithelial sites lending weight to their role as first-line effectors. Individual T cell receptor (TCR) variable region δ (Vδ) gene segments are associated with distinct ligand recognition and anatomical location. The positioning of these γδ T cells suggests a direct role of the TCR in each of these locations. The TCR may even be involved in retaining the cell at these locations (49). Thus, γδ T cells are usually categorized into two main types based on Vδ region: Vδ1 and Vδ2. In humans Vδ1 cells usually localize to tissues and are the main TCR type in the gut and skin. Some tissues contain highly specialized Vδ1 cells that are not found anywhere else in the body. For example, Vγ3Vδ1 skin dendritic epidermal T cells (DETC) arise exclusively in the epidermis, and Vγ5Vδ1 cells are only found in the intestinal epithelium. Vδ2 make up the largest population of γδ T cell family in the circulation of humans. The Vδ2 chain preferentially pairs with the Vγ9 (called Vγ2 in an alternative nomenclature) chain (50). These Vγ9Vδ2 cells comprise between 70 and 90% of the peripheral blood γδ T cell population. Although they make up less than 5% of total blood lymphocytes in healthy individuals, they can expand rapidly, up to 60% of peripheral blood lymphocytes, in certain infectious diseases due to their unique ligand recognition (51). This Vδ2 subtype is also responsible for the majority of the expansion in γδ T cells after BCG stimulation (45).

γδ T cells are involved in the first line of defense to a number of diseases, including cancer, bacterial infections, and viral infections. Studies have demonstrated their rapid activation and cytotoxicity to various viruses, including cytomegalovirus (CMV) (52, 53), influenza A virus (54–56), human immunodeficiency virus (HIV) (57–59), hepatitis B and C viruses (HBV and HCV) (60–62), Epstein Bar Virus (EBV) (63) and severe acute respiratory syndrome (SARS) virus (64), as reviewed by others (50, 51, 65–69). Additionally, γδ TCR knockout mice show an increase in viral titer or reduced survival when infected with West Nile virus or vaccinia virus (70, 71). After the 2003 SARS outbreak Poccia et al., evaluated lymphocytes in the circulation of survivors three months after initial infection. Interestingly, the number of αβ T cells did not differ from that of healthy uninfected subjects, but the numbers of Vδ2 T cells were substantially higher (64). This expansion was associated with higher anti-SARS-CoV immunoglobulin G (IgG). *In vitro* experiments showed that stimulated Vδ2 cells could kill cells infected with SARS-CoV, and that IFN-γ was involved in this response (64). Consequently, it is highly likely that γδ T cells could also be involved in the protective immune response to SARS-CoV-2.

Very few studies have investigated γδ T cells in SARS-CoV-2 infections, and the majority of information is in the context of severe disease. Laing et al., evaluated peripheral blood from hospitalized patients and showed lymphocytes were depleted in COVID-19 disease, the lymphocytes present were hyperactivated, whereas DC and monocyte functions were dampened. The drop in lymphocytes included γδ T cells, which were highly reduced in the circulation compared with healthy controls, especially the Vδ2 subset. This has also been reported by other studies (72–74). Lei et al., showed that the γδ T cells remaining in the blood had a CD25+ activated phenotype, although the very early activation marker CD69 did not increase compared to healthy controls, which the authors suggested may be because this marker was expressed earlier in infection (72). Notably, PD-1 expression did not change in these γδ T cells compared with controls, which suggests they were not exhausted. This contrasts with the finding that CD8+ cells showed heightened expression of both PD-1 and TIM3 related to disease severity, indicating a more exhausted phenotype in these cells as disease advances (75). Lastly, Lei et al., showed a dramatic increase in the proportion of γδ T cells co-expressing CD4, suggesting a role for this cell type, which is typically low in humans in homeostasis. Odak et al., showed a striking reduction in effector memory cells within the γδ T cell population, and an increase in naïve cells, and suggested that the effector memory γδ T cells may be recruited to the lungs. They also theorized that the reappearance of effector cells in the blood was associated with recovery from COVID-19 (74).

Many features of γδ T cells make them promising players in the SARS-CoV-2 response, including their key role in immunosurveillance of mucosal and epithelial barriers, their recognition of viral entry *via* a number of different pathways, and their functional responses that can act to kill virally infected cells as well as their ability to stimulate the adaptive immune system.

### γδ T Cell Recognition of Viral Infection

The mechanisms behind γδ T cell recognition of viral infections like SARS-CoV-2 are not as clearly understood as other cell types. γδ T cells use many different pathways to recognize foreign antigens and stress signals, and it is likely that different combinations of these pathways work synergistically in distinct viral infections to initiate and amplify responses. The main pathways include toll like receptors (TLRs), the γδ TCRs, and natural killer-like receptors.

γδ T cells express a variety of TLRs which bind to pathogen associated molecular patterns (PAMPs). Of particular importance are TLR 2 and 4 expressed on their cell membrane, which can recognize viral glycoprotein and glycolipids, as well as TLR 3 and 7, expressed on endosomes, which recognize viral RNA (76, 77). The binding of TLRs to PAMPs induces transcription factor upregulation, leading to pro-inflammatory cytokine production. The synergistic effects of TCR and TLR stimulation has been demonstrated *in vitro* by Wesch et al., where IFN-γ production in response to direct TCR stimulation is dramatically increased when TLR 3 is also stimulated with a synthetic analogue of its natural PAMP (78). TLR recognition of SARS-CoV-2 glycolipids, glycoprotein and RNA is likely a vitally important step in this immune response.

The Vδ2 TCR can recognize small phosphoantigens in a way that is unique, and likely responsible for its prolific responses in cancer, mycobacterial infection, and BCG vaccination. The first small phosphoantigen found to stimulate Vδ2 cells was a pyrophosphate intermediate of the mevalonate isoprenoid synthesis pathway, isopentenylpyrophosphate (IPP) (79). This pathway exists in all mammalian cells, and during normal physiological conditions, IPP is at a low concentration inside the cells and does not cause activation of Vδ2 cells. However, disruptions to the mevalonate pathway caused by a number of events, including dysregulated metabolism in tumors, pharmacological interference, or infections results in increases in intracellular IPP. Above a certain threshold IPP bound to the intracellular portion of butyrophilin-3A1 (BTN3A1) and BTN2A1 induces a conformational change that allows interaction of BTN2A1 with the Vγ chain of the TCR and likely also allow BTN3A1 to interact with the Vδ chain (80–82). Other small phosphoantigens that stimulate the BTN3A1 conformation change and subsequent Vδ2 TCR responses have now been identified. The most significant of these is produced by mycobacteria, including BCG, called (E)-4-hydroxy-3-methyl-but-2-enyl pyrophosphate (HMBPP). Microbial HMBPP, an intermediate of the MEP/DOXP pathway, has been found to activate Vδ2 cells with a potency 30000 times that of IPP (83).

Studies have suggested that the phosphoantigen/BTN mechanism of Vδ2 TCR activation may also have a role in viral infections, in addition to its importance in mycobacteria and cancer. Blocking the mevalonate pathway upstream with mevastatin, and therefore halting IPP synthesis, prevented the activation and proliferation of Vδ2 cells in an *in vitro* EBV infection (63). A similar outcome was seen when the mevalonate pathway was blocked in influenza A virus infection, where Vδ2 IFN-γ production was significantly reduced (54). It is currently unknown to what extent this pathway is active in SARS-CoV-2 infections. However, as it is likely responsible for much of the Vδ2 cell expansion after BCG, it is an important mechanism in the development of BCG primed anti-viral responses. Unlike Vδ2 cells, the Vδ1 TCR does not recognize phosphoantigen/BTN, and therefore Vδ1 cells proliferate less in response to BCG stimulation, although they are able to recognize BCG infected cells through the recognition of mycobacterial lipids on CD1. Recognition of CD1 in the context of viral infection is less understood, as there are no known virus specific lipids that exist in large enough quantities to be expressed on CD1. However, there is evidence that lipids derived from the host are presented on CD1 and can stimulate NK cells in viral contexts (84). The differentiation of CD1 displaying host lipid in homeostasis in comparison to viral infection, where substantial relocation of endosomal CD1 occurs has been hypothesized to mediate this stimulation (85). Some viruses, including Kaposi sarcoma associated herpesvirus (KCHV) and HIV actively induce the internalization of CD1, signifying CD1 presentation to NK cells or γδ T cells may contribute to protection (85).

In addition to TLRs and TCRs γδ T cells express other receptors, several of which are likely to be important in the recognition of viral infection, including NK type receptors (NKRs), DNAX Accessory Molecule 1 (DNAM1), and the Natural Cytotoxicity receptors (NCRs) NKp30, NK44 and NKp46. This review focuses on the NKR natural killer group 2-member D (NKG2D) only, as other NKRs have been recently reviewed by Caron et al. (69). NKG2D is an activating C-type lectin originally found on Natural Killer cells, but also highly expressed on both Vδ1 and Vδ2 T cells. It recognizes MHC class I polypeptide-related sequence A and B (MICA and MICB) and UL16 binding proteins (ULBPs). MICA, MICB and ULBPs can be expressed by the majority of cells, but are normally in very low abundance. Expression of these ligands is induced as part of the DNA damage response used by cells after stresses such as infection or malignant transformation (86). Once induced, their interaction with NKG2D on γδ T cells can assist activation and produce a powerful cytotoxic response. It is currently unknown the extent to which SARS-CoV-2 infection upregulates NKG2D ligands, however many ligands have been found to be upregulated on virally infected cells (69). For example, CMV infected cells have been shown to upregulate MICA and ULBP1-3 (87); EBV infected cells can upregulate MICA, MICB and ULBP4 (88, 89); and cells infected with either influenza A or Sendai virus can upregulate MICB (90). Blockade of NKG2D can also lead to a reduction in γδ T cell anti-viral responses (63).

### γδ T Cell Responses to Viral Infection

γδ T cells can mediate the killing of virally infected cells through a number of mechanisms. These include directly killing infected cells *via* cytotoxic molecules, and expression of membrane bound TNF-family members FasL and tumor-necrosis factor-related apoptosis-inducing ligand (TRAIL), as well as indirectly *via* the production of pro-inflammatory cytokines, and assisting in DC maturation to stimulate the adaptive immune system. These responses are important in the defense against SARS-CoV-2 infection and COVID-19 disease progression (91).

γδ T cells can secrete cytotoxic granules containing granzymes, perforin, and granulysin. These molecules have various effects on target cells that promote cell death. Perforin is able to form pores in target cell membranes, disrupting the osmotic balance, leading to an influx of Ca+ ions present at the immune synapse and pro-apoptotic signaling. Perforin also allows entry of granzymes. Granzyme B directly cleaves proteins involved in the caspase pathway, resulting in caspase-mediated apoptosis. It can also initiate the mitochondrial cell death pathway by cleaving BH3 interacting-domain death agonist (BID) (92). Granulysin can cause cell death in similar ways to granzyme B, and can also interfere with the target cell’s endoplasmic reticulum, which leads to pro-apoptotic signaling. Additionally, it has recently been shown that the 15kDa isoform of granulysin produced by γδ T cells, previously thought to be an inert precursor to the 9kDa isoform, can actually cause the migration and maturation of DCs (93). Other Granzymes that have been shown to kill virally infected cells in animal and *in vitro* models include granzyme M, H and K (94–96). Of interest, these lesser known granzymes may be able to inhibit viral replication by directly cleaving viral proteins, without necessarily killing the host cell, as exemplified by Granzyme M in a murine model of CMV infection (97).

γδ T cells can produce proinflammatory cytokines in response to viral recognition, including IFN-γ and TNF-α (98). These two cytokines trigger a multitude of pathways in target cells that can ultimately lead to the inhibition of viruses at all stages of their replication: viral entry, viral protein synthesis, viral assembly, and viral release, as recently reviewed (69). Many γδ T cells produce multiple pro-inflammatory cytokines simultaneously, which have synergistic effects on virally infected cells, and are particularly effective for viruses that have evolved escape mechanisms from one or many of the cytokine-induced pathways.

## BCG Stimulation of γδ T Cells to Combat Non-Tuberculosis Diseases

BCG stimulation of the immune system to target diseases other than TB is not a new concept, and has in fact been used for many decades before it was known how BCG could influence cells of the innate immune system, including γδ T cells. BCG has been used as a first line treatment for non-invasive bladder cancer since the 1970s, and can out-perform chemotherapeutic agents (99). BCG can also be used in the treatment of inoperable cutaneous melanoma (100–103). Studies have provided evidence that Vδ2 cells are contributing, at least in part, to BCG-induced regression of cancer cells, with BCG injections causing infiltration of Vδ2s into tumors and IFN-γ production (104). Other mycobacteria preparations are also in the process of commercialization, including IMM-101, an attenuated preparation of *Mycobacterium obuense*, which when used in combination with the first line treatment for inoperable pancreatic ductal adenocarcinoma (PDAC), the overall survival of patients improves (105).

BCG has many antigens that are potent stimulators of the immune system, and γδ T cells in particular. For example, BCG has a variety of cell wall lipids and proteins that are recognized by TLRs. Lipids from internalized BCG are also known to be displayed on CD1 molecules, that may be recognized by Vδ1 cells. As mentioned earlier, mycobacteria also produce the small phosphoantigen HMBPP, which potently stimulates Vδ2 TCRs. Therapeutics have been developed to specifically target this activation pathway using synthetic HMBPP and similar analogues, like Picostim (106, 107), as well as nitrogen-containing bisphosphonates (NBPs), which block the mevalonate pathway, leading to IPP accumulation (108, 109). Tu et al., expanded Vδ2 cells *in vitro* with the NBP pamidronate (PAM) and injected them into influenza infected humanized mice, demonstrating an improvement in disease severity and control of viral replication (110). Studies have also shown the NKG2D ligand MICA to be upregulated on epithelial and DCs after *M. tb* in humans (111), and mice NKG2D ligands Rae-2 and MULT1 are upregulated after BCG infection in the murine model (112).

**Figure 1:**
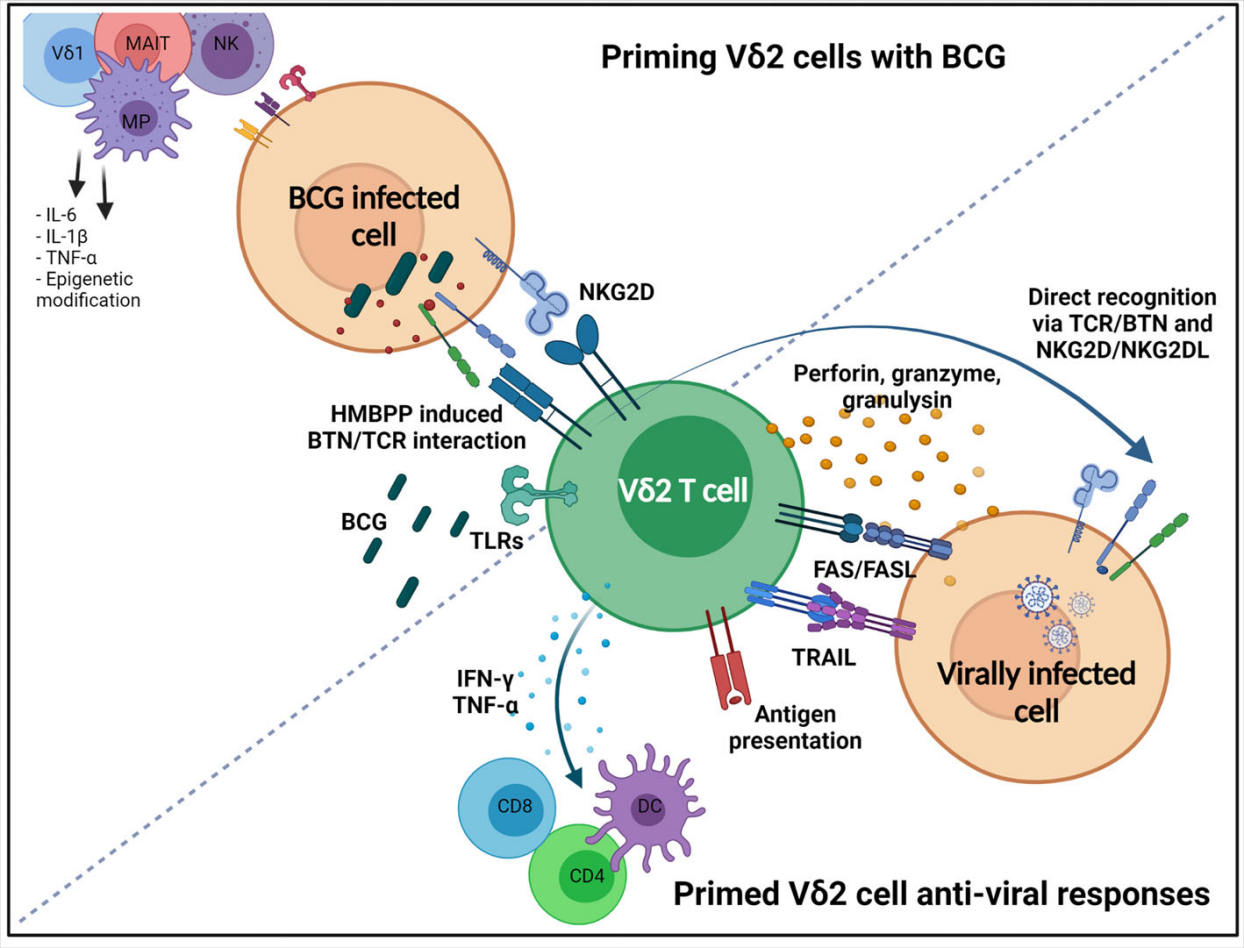
Priming Vδ2 cells with BCG, and subsequent non-specific anti-viral responses. Vδ2 T cells are activated after BCG vaccination through a number of mechanisms. HMBPP produced by BCG infected host cell causes conformational changes on intercellular domains of butyrophilin (BTN) molecules, such as BTN3A1 and BTN2A1, which allows the extracellular domain to interact with the Vδ2 TCR. Mycobacteria have been shown to induce the expression of NKG2D ligands on cells which can activate Vδ2 cells through NKG2D. Vδ2 cells have many TLRs that can recognize BCG PAMPs. Non-specific responses induced that have anti-viral activity include directly killing infected cells through the secretion of cytotoxic granules containing perforin, granzymes and granulysin, or initiation of death-inducing pathways, FASL and TRAIL. They can also indirectly contribute to killing through the production of pro-inflammatory cytokines TNF-α and IFN-γ inducing the maturation and migration of DCs, leading to induction of the adaptive immune system. Vδ2 cells may also recognize virally infected cells directly *via* NKG2D and Vδ2 TCR. Infected cells can upregulate NKG2D ligands (e.g. MICA, MICB), and can have altered metabolisms, which induces conformation changes to BTN molecules. Created with BioRender.com.

Therefore, BCG can stimulate γδ T cell activation through a variety of pathways, many of which are still unknown, and these can have synergistic effects on transcription to amplify anti-viral responses. Anti-viral γδ T cells responses that may be induced by BCG include the production of cytotoxic molecules, including granzyme B, granulysin and perforin (113); inflammatory cytokines, including IFN-γ and TNF-α (23); and the upregulation of death receptor ligands (69). Activated γδ T cells can also enhance the maturation and migration of DCs and present antigens themselves, thereby stimulating the adaptive immune system. This is summarized in **Figure 1**. Although this review is focused on γδ T cells due to their potential in COVID-19, BCG also impacts biological pathways of other cells of the immune system, as already discussed, including macrophages, NK cells, and MAIT cells, inducing epigenetic modifications to genes such as IL1β, TNFα, TLR4 and IL6, marking these cytokines as important and allowing for their rapid upregulation (44). Taken together, BCG are able to activate γδ T cells in similar ways to viral infections, and induce the production of molecules that are critical to the anti-viral response. Therefore, it is likely that priming γδ T cells with BCG can actively contribute to SARS-CoV-2 control and moderate the severity of the COVID-19 disease.

## Concluding Remarks

The BCG vaccine is affordable, commonplace, and non-specific. This makes it a rapid tool to implement in a pandemic such as COVID-19. Although we are only beginning to understand the innate mechanisms behind BCG’s broad protection, its impact on non-tuberculosis morbidity and mortality has been noted for a century (9). BCG vaccination can expand and prime innate and effector cells, including γδ T cells. γδ T cells are of particular interest, as BCG vaccination can induce them to direct potent anti-viral responses against infected cells, as well as stimulate the adaptive immune system. They have also been shown to be activated and not exhausted after COVID-19 infection. However, we need to remain aware of the vital role BCG already has in protecting against TB, particularly in infants in LMICs. Neonatal BCG vaccination remains a crucial component of TB control, and any delay to vaccination, such as that observed by BCG shortages in the past years, can have significant impacts on TB meningitis rates (114) and would be a major setback to global TB strategies. Any approach using BCG as a tool against COVID-19 should first prioritize BCG vaccines where they are needed most in LMICs with a high incidence of TB.

Considerations should also be made to the target age group and impacts of boosting and revaccination. BCG vaccination in the elderly has been shown to help protect against respiratory diseases, like COVID-19, indicating that BCG can also impact the innate immune system later in life (14). However, the efficacy of using BCG vaccination in adults to control TB varies widely (25). Vaccinating adolescents could conceivably have dual effects reducing the transmission of SARS-CoV-2 and *M. tb* (26). Using this dual strategy could have the greatest impact on reducing morbidity. The efficacy of BCG vaccination also varies globally, thought to be due to a number of factors including strains used, genetic and socio-economic differences, as well as interference *via* prior mycobacterial exposures, called masking and blocking. These are all factors that need to be considered in any BCG strategy to combat COVID-19 as they may impact how long non-specific protection lasts and as well as the requirement for boosting vaccinations.

Countries where TB rates are high often coincide with countries that have seen a delay in their antigen specific SARS-CoV-2 vaccine roll-out, and therefore are likely to be the countries where variants have full rein to develop. This last year has seen the rapid spread of variants across the world, and further mutations are expected to threaten the protection afforded by the current vaccines. BCG vaccination may provide a measure of protection independent of specific viral antigens, and therefore is unlikely to provide any selection pressure for new mutations, and is in fact likely to help control against new variants. If studies show BCG provides protection from COVID-19, a well-considered BCG strategy could contribute to the global effort against both COVID-19 and TB.

## Data Availability Statement

The original contributions presented in the study are included in the article/supplementary material. Further inquiries can be directed to the corresponding author.

## Author Contributions

AM designed, wrote, and revised the manuscript. AW, SS, and MB-S revised and edited the manuscript. All authors contributed to the article and approved the submitted version.

## Funding

This work was supported by a Public Health England PhD studentship and the Institute for Cancer Vaccines and Immunotherapy (Registered Charity Number 1080343).

## Conflict of Interest

The authors declare that the research was conducted in the absence of any commercial or financial relationships that could be construed as a potential conflict of interest.

## Publisher’s Note

All claims expressed in this article are solely those of the authors and do not necessarily represent those of their affiliated organizations, or those of the publisher, the editors and the reviewers. Any product that may be evaluated in this article, or claim that may be made by its manufacturer, is not guaranteed or endorsed by the publisher.
